# Assessing interpersonal violence involvement in psychiatric patients: exploratory findings on the Interpersonal Violence Questionnaire (IVIQ)

**DOI:** 10.1186/s12888-026-08597-7

**Published:** 2026-09-04

**Authors:** Paula Kittelmann, Claudia Helmert, Nicolai Hojka, Jana Karp, Melanie Pouwels, Anita Scheuermann, Jakob Steglich, Silvia Krumm, Georg Schomerus

**Affiliations:** 1 Department of Psychiatry and Psychotherapy, University of Leipzig, Leipzig, Germany; 2 Department of Psychiatry and Psychotherapy II, Ulm University, Ulm, Germany; 3 Vorurteilsfrei e.V., Leipzig, Germany

**Keywords:** Mental health service users, Severe mental illness, Victimization, Perpetration, Violence, Self-report, Trauma-informed care, Psychiatric assessment

## Abstract

**Background:**

Despite the high rates of both violence victimization and perpetration in people experiencing mental illness, standardized screening tools in research and for routine psychiatric assessment are lacking, underscoring the need for feasible, standardized assessment approaches.

**Methods:**

A sample of adult psychiatric in- and out-patients at six German hospitals was surveyed, using the Interpersonal Violence Questionnaire (IVIQ), a 26-item self-report checklist assessing experiences of physical violence, sexual violence, and threats, both as victimization and perpetration, across the past 12 months and lifetime. Exploratory analyses included prevalence estimates, scoring characteristics, floor effects, inter-item associations, temporal stability, and associations with social desirability.

**Results:**

In 479 patients, lifetime sexual victimization and 12-month violence threat, as well as being pushed, were most frequent. IVIQ sum scores showed strong floor effects. Reports of victimization and perpetration frequently co-occurred; descriptive inter-item correlations were low to moderate. Temporal consistency varied across items, with lower stability for low-prevalence behaviors. Higher social desirability predicted lower 12-month perpetration reporting.

**Conclusions:**

The IVIQ may provide a feasible, structured self-report approach for routine assessment of interpersonal violence involvement in psychiatric populations. Different scoring approaches may support descriptive assessment of cumulative burden and distinct forms of interpersonal violence involvement. Future versions should broaden the assessment of psychological violence. Associations between social desirability and reporting underline the importance of sensitive implementation, trauma-informed assessment practices, and cautious interpretation of self-reported violence experiences.

**Trial registration:**

This study is registered in the German Clinical Trial Register (DRKS) and the WHO International Clinical Trials Registry Platform (ICTRP) under registration number DRKS00032041 (Date of registration: 20th of July 2023).

**Supplementary Information:**

The online version contains supplementary material available at https://doi.org/10.1186/s12888-026-08597-7.

## Introduction

Despite its importance, research on interpersonal violence involvement among people with mental illness remains limited or is heterogeneous due to methodological limitations. Existing studies vary widely in definitions and operationalization of violence, survey periods, and sample characteristics, resulting in prevalence estimates that make it difficult to compare across studies [1–3]. Estimates based on police or health records are likely to underestimate the prevalence of interpersonal violence [4], as a substantial number of incidents are not reported. Population- or clinic-based self-report surveys help to provide additional insight into experiences that might otherwise remain undocumented [5], but are often restricted by the use of single-item measures or by their focus on certain forms of violence only, such as intimate partner violence or adverse childhood experiences [6].

Contrary to common stigmatizing beliefs, it is well established that people with severe mental illness (SMI) are more likely to experience victimization than perpetration of violence [1, 7]. However, labeling individuals as either victims or perpetrators of violence might not sufficiently reflect the range of interpersonal violence involvement among people with mental illness. Studies examining the overlap between victimization and perpetration suggest that past victimization plays a significant role in subsequent violent behavior, and vice versa [8–10]. This highlights the need for structured and clinically feasible approaches to assessing both victimization and perpetration in psychiatric populations [11].

Although several standardized instruments are available to assess specific forms of violence or trauma (e.g., adverse childhood experiences, childhood maltreatment, or intimate partner violence) [12–14], there is currently no generally accepted screening instrument for the broader assessment of interpersonal violence involvement—including both victimization and perpetration across different contexts—in psychiatric clinical settings [15–17], despite the high prevalence, the implications for therapy and recovery, and the risk of future violence victimization and perpetration [4, 18–20]. Structural barriers, such as limited consultation time, clinicians’ discomfort with sensitive topics, and worries about follow-up procedures, might hinder the routine assessment of experiences of interpersonal violence in psychiatric care [21, 22]. Standardized screening tools are already commonly used in domains such as substance use and trauma assessment [23], suggesting utility for a structured assessment of interpersonal violence involvement in psychiatric settings.

The present study contributes to research on interpersonal violence in psychiatric populations by introducing a brief self-report checklist for the structured assessment of patient-reported victimization and perpetration across different violence domains and time frames. We introduce the Interpersonal Violence Questionnaire (IVIQ), a brief self-report checklist intended to support more standardized assessment of interpersonal violence experiences, including both victimization and perpetration.

Victimization is defined as self-reported exposure to acts of violence by others, and perpetration is defined as self-reported commission of violent acts against others. Violence is conceptualized as the occurrence of specific interpersonal behaviors (physical, sexual, or threat-related acts), based on definitions from the WHO Report on Violence and Health [24], and independent of their psychological impact. This distinguishes violence involvement from trauma exposure, which refers to the subjective psychological response to potentially distressing events [25]. This distinction is particularly important in clinical populations, where not all experiences of interpersonal violence are necessarily appraised or reported as traumatic. The operationalization of physical violence as observable and definable by certain acts is frequently used in epidemiological and psychosocial research and was thus used to facilitate a structured and comparable assessment [26, 27]. Interpersonal violence involvement was assessed as self-reported incidents, consistent with a social-scientific conceptualization of interpersonal violence rather than a legal definition. Development of this instrument was part of a participatory research project on experiences of violence among people with mental illness [28].

## Methods

### Ethics and trial registration

The study was approved in accordance with the Declaration of Helsinki by the Ethics Committee at Leipzig University (ID: 439-21-ek), the Ethics Committee of the State Chamber of Physicians of Saxony (EK-BR-65/42 − 1) as well as the Ethics Committee of Ulm University (ID: 454 − 23/MO/mm).

This study is registered in the German Clinical Trial Register (DRKS) and the WHO International Clinical Trials Registry Platform (ICTRP) under registration number DRKS00032041 (Date of registration: 20th of July 2023).

### Informed consent and consent for publication

Each potential study participant was verbally and in writing informed about the aims, procedures, data policies, and potential risks of the study. A copy of the information sheet remained with the study participants. Potential participants were informed about the pseudonymous procedure; each participant generated their own cipher on the informed consent form. In the survey itself, no personal data was collected, just the cipher. Informed consent was obtained before participants were handed the survey. Study participation could be refused or withdrawn at any time without any negative consequences. Consent for publication was not applicable in this study.

### Sample, recruitment, and data collection

To recruit a broad sample of people receiving psychiatric in-patient or out-patient treatment at a psychiatric hospital, we conducted a survey among all eligible patients on a specified date in six different psychiatric hospitals across urban and rural regions in Germany (for the list of clinics, see Supplementary material 1). No forensic psychiatric facilities were included in the present study. Recruitment across multiple sites was chosen to capture patient-reported interpersonal violence involvement across different psychiatric care settings. Inclusion criteria were legal age (18 years) and German language proficiency (questionnaires were only presented in German). Exclusion criteria were dementia, intellectual impairment, current psychotic symptoms, and being currently in isolation or fixation. Patients with psychotic symptoms in full or partial remission were not excluded. All patients who met the predefined eligibility criteria, as determined by the responsible clinical staff, and who were present on a ward or attending an outpatient clinic on the predefined recruitment day were approached by study staff. Patients who agreed to participate and gave informed consent completed the questionnaires via tablet or paper and pencil in a group setting or their private room in the clinic. Study staff were available for inquiries or to provide help at all times. Data were collected and managed using REDCap (Research Electronic Data Capture) [29]. Paper-and-pencil data were later transferred to REDCap by the study staff. Participants consenting to follow-up were contacted by email after 4–5 weeks to examine the temporal consistency of reported events. Participants who didn’t answer or didn’t complete the second survey within a period of 1 week were then sent two reminders via email. After that, if they did not complete the survey, they were not contacted again.

### Survey

*Sociodemographic variables* included age, gender, socioeconomic status, sexual orientation, and living situation, as well as immigration experience as first or second generation, and experience with discrimination due to race and other factors.

*KSE-G.* Part of the survey was a scale for assessing the social desirability trait (“Kurzskala Soziale Erwünschtheit-Gamma” (KSE-G)) [30].

*IVIQ.* In this study, interpersonal violence involvement refers to engagement in discrete acts of interpersonal violence, operationalized as self-reported victimization and/or perpetration.

To assess interpersonal violence involvement, including victimization and perpetration, we used a list of items provided by Harris et al. [31], who adapted parts of the interview that were established in the MacArthur Risk Study, the MacArthur Community Violence Screening Instrument (MCVSI) [3, 31–33]. The use of structured, behavior-specific questions has been recommended compared to the use of broad or ambiguous definitions [26].

The IVIQ consists of 26 questions about victimization and perpetration (listed in Table 1; for the German version, see Supplementary Table 2). Interpersonal violence involvement was assessed in parallel for victimization and perpetration, reflecting that both represent distinct behavioral roles that may co-occur but are not interchangeable constructs. Items were designed to capture distinct acts of violence as salient events rather than a latent construct, consistent with checklist-based measurement approaches [34–36].

**Table 1:** IVIQ items, prevalence estimates, floor effects, missing responses, and mean inter-item φ coefficients

		Item	Type		“yes” n (%)	“no” n (%)	“prefer not to say” n (%)		missings n (%)		mean inter-item φ
Past 12 months	(1)	vio_1. Has someone thrown something at you?	vict	physical	78 (16.3)	385 (80.4)		7 (1.5)		9 (1.9)	0.32
		vio_2. Have you thrown something at someone?	perp		32 (6.7)	434 (90.6)		9 (1.9)		4 (0.8)	0.29
		vio_3. Has anyone pushed, grabbed or shoved you?	vict		127 (26.5)	336 (70.2)		9 (1.9)		7 (1.5)	0.32
		vio_4. Have you pushed, grabbed or shoved anyone?	perp		89 (16.7)	386 (80.6)		7 (1.5)		6 (1.3)	0.22
		vio_5. Has anyone slapped you?	vict		68 (14.2)	393 (82.1)		10 (2.1)		8 (1.7)	0.32
		vio_6. Have you slapped anyone?	perp		33 (6.9)	430 (89.8)		11 (2.3)		5 (1.0)	0.28
		vio_7. Has anyone kicked, bitten or choked you?	vict		69 (14.4)	392 (81.8)		10 (2.1)		8 (1.7)	0.32
		vio_8. Have you kicked, bitten or choked anyone?	perp		22 (4.6)	44 (92.7)		8 (1.7)		5 (1.0)	0.29
		vio_9. Has anyone hit you with a fist or beaten you up?	vict		48 (10.0)	418 (87.3)		6 (1.3)		7 (1.5)	0.32
		vio_10. Have you hit anyone with a fist or beaten up anyone?	perp		28 (5.9)	436 (91.0)		10 (2.1)		5 (1.0)	0.32
		vio_11. Has anyone threatened you with violence?	vict	thr	135 (28.2)	317 (66.2)		16 (3.3)		11 (2.3)	0.29
		vio_12. Have you threatened anyone with violence?	perp		53 (11.1)	416 (86.9)		6 (1.3)		4 (0.9)	0.29
	(2) higher severity	vio_13. Has anyone tried to physically force you to have sex against your will?	vict	sex	59 (12.3)	400 (83.5)		15 (3.1)		5 (1.0)	0.24
		vio_14. Have you tried to physically force anyone to have sex against their will?	perp		1 (0.2)	474 (99.0)		3 (0.6)		1 (0.2)	0.19
		vio_15. Has anyone threatened you with a knife, gun, or any other weapon?	vict	thr	49 (10.2)	425 (88.8)		2 (0.4)		3 (0.6)	0.36
		vio_16. Have you threatened anyone with a knife, gun, or any other weapon?	perp		15 (3.1)	459 (95.8)		2 (0.4)		3 (0.6)	0.28
		vio_17. Has anyone fired a gun at you or used a knife or any other weapon on you?	vict	physical	20 (4.2)	451 (94.2)		4 (0.8)		4 (0.8)	0.27
		vio_18. Have you fired a gun at someone or used a knife or other weapon on them?	perp		6 (1.3)	468 (97.7)		3 (0.6)		2 (0.4)	0.20
		vio_19. Have you hurt somebody really badly?	perp		29 (6.1)	437 (91.2)		9 (1.9)		4 (0.8)	0.30
Lifetime		vio_20. Has anyone ever tried to physically force you to have sex against your will?	vict	sex	207 (43.2)	250 (52.2)		19 (4.0)		3 (0.6)	0.13
		vio_21. Have you ever tried to physically force anyone to have sex against their will?	perp		10 (2.1)	459 (95.8)		4 (0.8)		6 (1.3)	0.11
		vio_22. Has anyone threatened you with a knife, gun, or any other weapon?	vict	thr	100 (21.9)	367 (76.6)		7 (1.5)		5 (1.0)	0.28
		vio_23. Have you threatened anyone with a knife, gun, or any other weapon?	perp		36 (7.5)	428 (89.4)		6 (1.3)		9 (1.9)	0.25
		vio_24. Has anyone fired a gun at you or used a knife or any other weapon on you?	vict	physical	48 (10.0)	418 (87.3)		5 (1.0)		8 (1.7)	0.26
		vio_25. Have you fired a gun at someone or used a knife or other weapon on them?	perp		22 (4.6)	446 (93.1)		5 (1.0)		6 (1.3)	0.24
		vio_26. Have you ever hurt somebody really badly?	perp		52 (10.9)	412 (86.0)		9 (1.9)		6 (1.3)	0.27

Nineteen items refer to interpersonal violence involvement in the past 12 months. A subset of seven items describing higher severity was also used to assess lifetime violence exposure. If participants answered “yes”, they were asked about how many times it happened, as well as who the perpetrator/victim was, and, in addition to the original interview, we added a question about the location of the incident. According to Harris et al. [31], we added the response option “Prefer not to say”. From this questionnaire, we calculated three different measures of prevalence: a 12-month prevalence based on all 19 items, a lifetime prevalence based on the seven “higher severity” items, and a “higher severity” 12-month prevalence using these same seven items, to allow for direct comparison between lifetime and 12-month prevalence. The categorization of “higher severity” items was based on an a priori distinction based on objective harm potential rather than subjective burden.

Items from the list provided were first translated into German by the study team [37]. We focused primarily on physical violence. However, we added an extra item to the original list to assess whether someone had threatened or been threatened by another person (i.e., threat of violence), in addition to the existing item “threatened with a weapon” from the original version. Past research considered that threats constitute a form of violence that occurs frequently [38]. The questionnaire ended with two open questions about further remarks regarding the violence involvement and/or the questionnaire. Answers were evaluated descriptively and are listed in Supplementary material 4.

The items and the structure of the questionnaire were then reviewed by a group of peer researchers with lived experience of violence and psychiatric treatment (Vorurteilsfrei e.V. Leipzig) and adjusted regarding linguistic sensitivity and accessibility. Finally, the questionnaire was pre-tested for comprehensibility, language appropriateness, question sensitivity, feasibility of administration, and average completion time among asound 30 patients on three wards of the Leipzig University Hospital Clinic and Polyclinic for Psychiatry and among volunteers from the workgroup with no defined mental illness and no connection to the research project. It was revised for clarity and readability and finalized based on their feedback.

### Analysis

#### Software

Statistical analyses were conducted using Stata 18 [39].

#### Scoring

For each participant, items assessing violence exposure in the past 12 months or lifetime were combined to derive dichotomous scores, indicating overall interpersonal violence involvement (any victimization and/or perpetration). Any participant reporting at least one act of violence was coded as “1” (yes), and participants reporting no acts were coded as “0” (no). This approach is consistent with previous studies [27, 31] and was intended to provide a pragmatic indicator of reported interpersonal violence involvement. Participants with incomplete responses across items were coded as positive if any act was reported; otherwise, they were coded as negative, resulting in no missing values for the final dichotomous indicators.

We also calculated separate sum scores of different acts of violence for perpetration and victimization across both reference periods, to examine the breadth of violent acts, as well as sum scores for reported frequencies of violent events [6].

Floor effects were examined [40, 41]. Pairwise Phi coefficients [42] were computed to explore co-occurrence. Internal consistency indices and factor-analytic approaches were not applied, as the IVIQ is designed as a behavioral checklist rather than a reflective scale. Content-related aspects were addressed through expert review, translation checks, and pretesting procedures [43].

A 2 × 2 table (victimization = 0/1 vs. perpetration = 0/1) for both timeframes (past 12 months and lifetime) was conducted. Phi coefficients were then calculated between these dichotomized subscales. This calculation is for descriptive and comparative purposes, not as tests of latent structures.

Acceptability was explored using participants’ free-text responses regarding the questionnaire and the topic of violence (Supplementary material 4), as well as patterns of item non-response.

#### Re-test and temporal stability

To assess the temporal stability of reported events, participants who completed both time points were included in the retest analysis. Because the IVIQ is a behavioral checklist assessing the occurrence of interpersonal violence rather than a stable psychological construct, the retest was intended to examine the temporal consistency of reported events rather than classical test–retest reliability. The retest interval of 4–5 weeks was chosen to ensure feasibility while allowing assessment of temporal stability. Percentage agreement and Intraclass Kappa Indices (ICC) for dichotomous response data were calculated for each item to explore temporal consistency of responses, as suggested by Fisher and colleagues [44]. The unit of analysis was the individual participant, with the two measurement occasions (complete T1 and T2 pairs) treated as fixed raters. Simple agreement (percentage of identical responses across timepoints) was additionally examined to provide a descriptive measure of consistency.

#### Social desirability

Associations between social desirability and reported victimization and perpetration were examined using binary logistic regression models for 12-month and lifetime prevalence indicators. Social desirability (continuous KSE-G sum score) was entered as an independent variable, adjusted for age and gender.

## Results

### Sample characteristics

Of the 987 patients considered eligible and approached across the participating sites, 493 participated (49.9%). 14 participants were excluded post-hoc due to early dropout and suspected psychotic symptoms. The final sample thus consisted of 479 participants. The mean age was 40.38 years (SD = 15.54, range 18–88). The sample included 193 men (40.3%), 252 women (52.6%), 20 participants identifying as “diverse” (4.2%), and 1 participant with no information on their gender (0.2%). 330 participants (68.9%) were inpatients, 149 participants (31.1%) visited a day clinic or were outpatients.

### Item prevalence

Prevalence rates per item, floor effects, and missing values are reported in Table 1. The most frequently reported form of victimization was lifetime sexual violence (vio_20, 43.2%), followed by the threat of violence in the past 12 months (vio_11, 28.2%) and being pushed/grabbed or shoved in the past 12 months (vio_3, 26.5%).

Missing data were below 5% across variables; therefore, no imputation was performed [45]. Given the low proportion of missingness, potential bias was considered limited, although it cannot be fully excluded.

### Prevalence of interpersonal violence involvement and sum scores

Using the dichotomous scoring approach, 44.1% of participants reported victimization in the past 12 months, whereas 51.1% reported victimization in their lifetime. 27.1% reported perpetration in the past 12 months, 16.7% in their lifetime (Table 2).

**Table 2:** Dichotomous indicators of reported victimization and perpetration

	Past 12 months (1)		Past 12 months (2)		Lifetime	
	vict	perp	vict	perp	vict	perp
Yes (n, %)	211 (44.1)	130 (27.1)	89 (18.6)	39 (8.1)	245 (51.1)	80 (16.7)
No (n, %)	268 (56.0)	349 (72.9)	390 (81.4)	440 (91.9)	234 (48.9)	399 (83.3)

Note. (1) includes all of the 12-month items (2), includes only the items of higher severity (vict.: vio_13, vio_15, vio_17; perp.: vio_14, vio_16, vio_18, vio_19) for comparability with the lifetime items

**Fig. 1:**
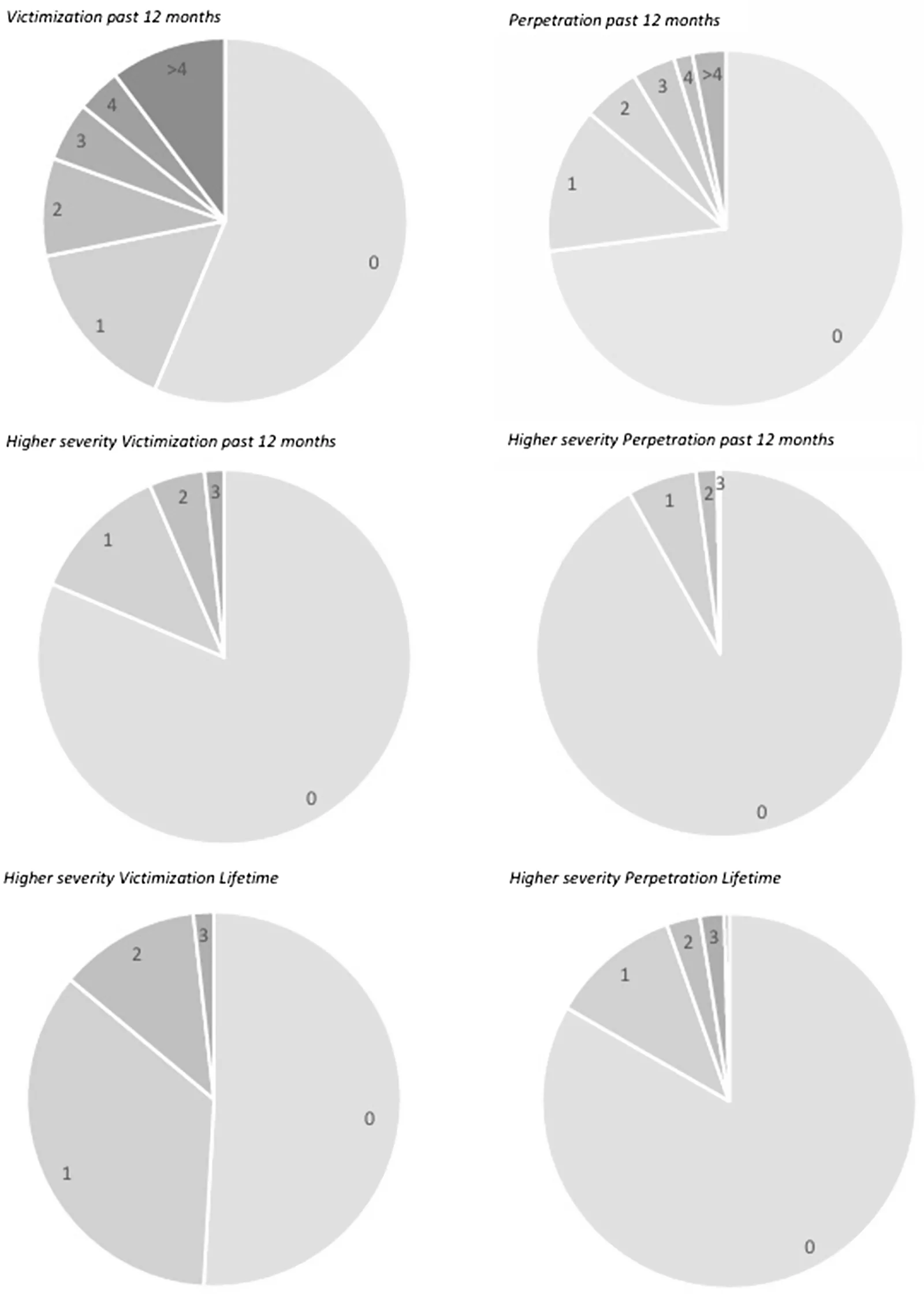
Distribution of the number of different IVIQ violence acts endorsed (sum score) for victimization and perpetration during the past 12 months and lifetime. Note. The cumulative exposure score represents the number of different IVIQ violence acts endorsed within each domain (victimization, perpetration, higher-severity victimization, and higher-severity perpetration). Thus, a value of 0 indicates that no act within the respective domain was endorsed, 1 indicates one endorsed act, 2 indicates two different acts, and so forth. The score reflects the number of different acts rather than their frequency. Categories >4 were combined because of small cell sizes *n* = 479

The distribution of sum scores, as shown in Table 3, reflects the number of different acts of violence (victimization and perpetration). Median sum scores were 0 across all scales (ranges from 0 to 9 and 0 to 10). Mean sum scores were low (M = 0.11–1.40, SD = 0.40–2.17). Figure 1 represents the distribution of the number of different acts of interpersonal violence.

**Table 3:** Sumscores across different types of violence in the 12-month and lifetime period for victimization and perpetration

Sumscore *n* (%)	12 months (1)		12 months (2)		lifetime	
	vict	perp	vict	perp	vict	perp
0	268 (55.9)	349 (72.9)	390 (81.4)	440 (91.9)	234 (48.9)	399 (83.3)
1	74 (15.5)	63 (13.2)	58 (12.1)	29 (6.1)	162 (33.8)	54 (11.3)
2	41 (8.6)	25 (5.2)	23 (4.8)	9 (1.9)	56 (11.7)	14 (2.9)
3	25 (5.2)	19 (3.9)	8 (1.7)	0 (0)	27 (5.6)	10 (2.1)
4	19 (3.9)	8 (1.7)	0 (0)	1 (0.2)	0 (0)	2 (0.4)
5	11 (2.3)	5 (1.0)	0 (0)	0 (0)	0 (0)	0 (0)
6	14 (2.9)	3 (0.6)	0 (0)	0 (0)	0 (0)	0 (0)
7	13 (2.7)	4 (0.8)	0 (0)	0 (0)	0 (0)	0 (0)
8	10 (2.1)	2 (0.4)	0 (0)	0 (0)	0 (0)	0 (0)
9	4 (0.8)	0 (0)	0 (0)	0 (0)	0 (0)	0 (0)
10	0 (0)	1 (0.2)	0 (0)	0 (0)	0 (0)	0 (0)

Note. Sumscore refers to the number of different types of interpersonal violence (1). includes all of the 12-month items (2), includes only the items of higher severity (vict.: vio_13, vio_15, vio_17; perp.: vio_14, vio_16, vio_18, vio_19) for comparability with the lifetime items

Frequency scores showed a highly right-skewed distribution. To account for the zero-inflated and skewed frequency distribution, zero-prevalence and frequency were reported separately. Quartile distributions are provided in Table 4. Missing frequency responses were coded as zero for descriptive analyses, resulting in higher zero-prevalence for frequency scores than incident sum scores. Extremely high values (e.g., 999) for the frequency questions were used by participants to indicate occurrences that were “too frequent to count”, following prior instructions provided by study staff, and were included in the fourth quartile.

**Table 4:** Sumscores for frequencies in quartiles across different types of violence in the 12-month and lifetime period for victimization and perpetration

		12 months (1)		12 months (2)		lifetime	
		vict	perp	vict	perp	vict	perp
No frequency/violence reported (frequency = 0) n (%)		284 (59.3)	362 (75.6)	406 (84.8)	448 (93.5)	274 (75.2)	412 (86.0)
Q1	n (%)	60 (12.5)	33 (6.9)	20 (4.2)	15 (3.1)	75 (15.7)	28 (5.9)
	min-max	1–2	1	1	1	1–2	1
Q2	n (%)	44 (9.2)	34 (7.1)	19 (4.0)	7 (1.5)	39 (8.1)	8 (1.7)
	min-max	3–4	2–3	2–3	2	3–5	2
Q3	n (%)	43 (9.0)	21 (4.4)	16 (3.3)	5 (1.0)	40 (8.4)	15 (3.1)
	min-max	8–32	4–7	4–7	3	6–45	3–10
Q4	n (%)	48 (10.0)	29 (6.1)	18 (3.8)	4 (0.8)	51 (10.7)	16 (3.3)
	min-max	> 36	> 8	> 9	> 4	> 50	> 12

Note. Summed frequency scores were categorized into quartiles across different types of interpersonal violence for victimization and perpetration in the 12-month and lifetime periods. Quartiles are reported only for participants who reported at least one act of victimization or perpetration; participants with no reported incidents (frequency = 0) are shown separately. In the fourth quartile, only the lower limit of the quartile was reported; outliers were included (1). includes all of the 12-month items (2), includes only the items of higher severity (vict.: vio_13, vio_15, vio_17; perp.: vio_14, vio_16, vio_18, vio_19) for comparability with the lifetime items

### Floor effects

The majority of items showed floor effects according to the 15% criterion [40]. The percentage of “no” responses was used here as a direct indicator of floor effects. For sum scores, a substantial portion of participants scored at the minimum (48.9–83.3%), indicating pronounced floor effects. Distributional indices additionally indicated strong positive skew across sum scores.

### Inter-item correlations

Average Phi coefficients per item are reported in Table 1 (for Pairwise Phi coefficients, see Supplementary Table 5). Due to low base rates, coefficients are interpreted descriptively. Within the 12-month reference period, higher correlations were observed among items assessing physical victimization (φ > 0.50). Threat-related items also showed associations with physical violence items. Within the lifetime reference period, weapon-related items showed comparatively stronger associations with one another.

These items also showed moderate descriptive associations with several other items (Table 1). No large associations were found for items assessing sexual violence. These items also showed substantial floor effects (Table 1).

### Correlation of reports of victimization and perpetration

Phi coefficients indicated moderate associations. Associations were stronger for past 12-month reports and small to moderate for “higher severity” or lifetime events (Table 5).

**Table 5:** Prevalence and association of victimization and perpetration in the past 12 months and lifetime

	Past 12 months (1)		Past 12 months (2)		Lifetime	
	Vict *n* (%)	Perp *n* (%)	Vict *n* (%)	Perp *n* (%)	Vict *n* (%)	Perp *n* (%)
Yes (%)	211 (44.1)	130 (27.1)	89 (18.6)	39 (8.1)	245 (51.2)	80 (16.7)
*Φ*	0.49		0.29		0.19	
*χ² (1*,*N = 479)*	114.65		40.2		17.52	
*p*	0.00		0.00		0.00	

Note. φ = Phi coefficient. χ² (1) = Chi-square statistic with 1 degree of freedom. p-Values < 0.001 are considered significant (1). includes all of the 12-month items (2), includes only the items of higher severity (vict.: vio_13, vio_15, vio_17; perp.: vio_14, vio_16, vio_18, vio_19) for comparability with the lifetime items

### Retest sample

Of all baseline participants, 283 consented to re-contact; 113 completed the retest 4–8 weeks after the first survey. The mean age was 38.6 years (SD = 13.6), and 35.4% were male, 54.9% female, and 8.0% diverse.

Temporal consistency of the 26 items was explored using test–retest analyses (Supplementary Table 3). ICC estimates ranged from near zero to 0.74 across items and should be interpreted cautiously given the low prevalence of several items, which showed little or no variance across measurements.

### Social desirability

The social desirability scale (KSE-G; range 0–24) showed a mean of 15.72 (SD = 4.12). The distribution deviated significantly from normality (Shapiro–Wilk test, *p* < .001). No significant gender differences were observed. Social desirability was associated with lower odds of reporting at least one perpetration of violence during the past 12 months (OR = 0.93, Table 6), but not with the overall victimization or lifetime prevalence indicators.

**Table 6:** Binary logistic regression analyses

Factors	Past 12 months				Lifetime			
	Vict		Perp		Vict		Perp	***p***
	**OR** **(95% CI)**	***p***	**OR** **(95% CI)**	***p***	**OR** **(95% CI)**	***p***	**OR** **(95% CI)**	
Social desirability (KSE-G)	0.99 [0.94, 1.04]	0.59	**0.93 ** **[0.88**,** 0.98]**	0.01	0.98 [0.93, 1.03]	0.34	0.95 [0.89, 1.02]	0.15
Female gender ^1^ (Ref. male)	0.95 [0.64, 1.42]	0.81	0.85 [0.55, 1.32]	0.48	**2.98 ** **[1.98**,** 4.48]**	< 0.001	**0.21** ** [0.12**,** 0.38]**	0.03
Age	**0.97 ** **[0.95**,** 0.98]**	< 0.001	0.99 [0.98, 1.01]	0.28	**0.97 ** **[0.96**,** 0.98]**	< 0.001	**0.98 ** **[0.96**,** 1.00]**	< 0.001

Note. OR = odds ratio; 95% CI = confidence interval. Victimization and perpetration coded as 0 = no, 1 = at least one experience

## Discussion

This study presents an exploratory evaluation of the IVIQ as a structured self-report checklist for assessing patient-reported interpersonal violence involvement. Findings suggest that the IVIQ may provide a feasible approach across psychiatric care settings. Observed prevalence patterns suggest comparability with previous research [46].

### Acceptability, burden, and feasibility of the IVIQ

Based on comments made by participants during on-site data collection, the administration of the IVIQ proved feasible in clinical settings, with only 10 participants dropping out due to emotional distress. Qualitative feedback indicated that participants found the topic of the questionnaire highly relevant (see Supplementary material 4). Both in- and outpatients were able to complete the instrument without extensive guidance, supporting its potential use as a feasible structured self-report checklist in psychiatric settings.

### Conceptual and methodological considerations in defining violence

In this study, we defined violence primarily as physical violence, with the use of structured, behavior-specific questions. The focus on physical violence was chosen to improve behavioral specificity and comparability across studies, while acknowledging that broader conceptualizations of violence should be considered in future research. Free-text fields were included to capture subjective interpretations of violence. Behavior-specific questions may facilitate recall and reporting of violence experiences [47].

The categorization of “higher severity” does not necessarily reflect subjective burden. Repeated exposure and polyvictimization may nevertheless render “less severe” acts clinically highly relevant. The label “higher severity” should be interpreted as an analytical category rather than an indicator of individual-level burden.

### Measurement model and psychometric implications

The IVIQ includes different scoring approaches, namely dichotomous prevalence indicators, cumulative exposure scores, and frequency-based descriptive indices [48], which allow descriptive examination of interpersonal violence involvement from multiple perspectives. To examine more than one act or form of violence is essential, especially in high-risk groups, and to account for the cumulative burden and the mental health issues that may be the consequence of such experiences [6, 49]. Frequency distributions were skewed and zero-inflated, which is expected when assessing rare events like interpersonal violence involvement [6, 50].

The IVIQ was conceptualized as a behavioral checklist rather than a latent construct measure. Therefore, internal consistency indices were not considered appropriate criteria.

Retest analyses provided information on the temporal consistency of reported events rather than on the stability of a psychological construct. Because the IVIQ assesses experiences of interpersonal violence, thus behavioral events, rather than stable traits, responses may legitimately differ across measurement occasions due to newly occurring events, changes in recall, or disclosure. Lower agreement estimates were primarily observed for rare behaviors and may therefore reflect both low base rates and possible changes in recall or event occurrence within the reference period [41, 51] rather than poor item performance.

### Victim-perpetrator-overlap

Assessing both victimization and perpetration enabled examination of their overlap within psychiatric populations. In this study, results indicate that individuals who experience violence victimization are at substantial risk of also perpetrating it, and vice versa. These findings have practical implications for clinical assessment routines and treatment planning.

### Social desirability and barriers to disclosing

The social desirability bias might lead to underreporting of interpersonal violence involvement, due to shame, stigmatization concerns, or fear of negative consequences [46, 52]. In the present study, higher social desirability reporting primarily affected overall reports of violence involvement during the past 12 months, particularly the aggregated perpetration measures.

Considering the “prefer not to say”-option as a hint to acceptability and barriers to disclosing some experiences, it is interesting to note that the item “Has anyone tried to physically force you to have sex against your will?” for both the past 12 months and lifetime shows a slightly higher percentage than the other items (3.1%, 4.0%, respectively). This hints at the sensitivity with which this type of experience of violence must be addressed.

### Participation and recall bias

Participation bias cannot be excluded. Experiences of violence may have influenced willingness to participate (out of interest or the wish to talk about experiences) as well as not to participate (e.g., due to shame or fear of distress). In addition, recall bias may affect retrospective reports, particularly for lifetime experiences, and represents a general limitation of self-report data in this population [53–55].

### Limitations

A number of methodological and conceptual limitations should be taken into account when interpreting the results.

*Criterion validity* was not examined due to the lack of validated German self-report instruments assessing violence in a comparable wider definition in this target group, instead of just assessing experiences of a certain form of violence, such as intimate partner violence [6].

*Sampling*. The study included psychiatric in- and outpatient populations with different diagnoses that are often underrepresented in violence research. The sample consists of individuals currently receiving psychiatric treatment, limiting generalizability to untreated populations. Moreover, the above-mentioned exclusion criteria are risk factors for victimization [56, 57]. For example, German language skills as an inclusion criterion excludes refugees with PTSD (post traumatic stress disorder) or other mental health problems, a group at increased risk of violence exposure and vulnerability [58].

*Definition of Violence*. Limiting experiences of violence in this survey to mostly acts of physical violence does not fully account for the complexity of experiences of violence or for how individuals perceive their own experiences. Comments from participants (Supplementary material 4) highlighted limitations on the definition of researcher-defined versus subjective concepts of violence.

For example, psychological violence was not systematically assessed. Participant feedback suggested that many individuals considered these experiences relevant (Supplementary material 4), indicating that future versions should broaden the operationalization of violence and be expanded to include various acts of violence depending on research interests. This also applies to sexual violence, which in the current IVIQ is represented by one behavioral item per timeframe and therefore does not capture the full spectrum of sexually coercive experiences.

*Timeframes*. Inclusion of both 12-month and lifetime reference periods allowed comparison with existing research [46]. However, some participants reported difficulties distinguishing between the two timeframes, suggesting the need for a revised layout or formatting. Moreover, limiting lifetime items to major events could underestimate violence involvement, as noted by participants (Supplementary material 4).

*Analyses*. Given the exploratory nature of some analyses, findings should be interpreted with caution. Pronounced floor effects limited the interpretability of some association and stability estimates. Missing data were expected due to the sensitivity of the topics in this survey, but no formal MCAR test (e.g., Little’s test) could be conducted due to the dichotomous nature of the items.

Furthermore, the retest interval cannot distinguish true changes in violence exposure from recall processes or response tendencies, which limits the interpretation of temporal stability estimates for behavioral event measures.

Taken together, the findings provide preliminary support for the feasibility of the IVIQ as a structured self-report approach that may support routine assessment of interpersonal violence involvement in psychiatric care.

## Implications

The IVIQ may support structured assessment of patient-reported interpersonal violence experiences in both psychiatric research and clinical care.

### Implications for future research

Future studies may benefit from multi-level scoring approaches depending on the research question.

Future versions of the IVIQ may benefit from expanded assessment of lifetime experiences. In addition, items addressing psychological violence and emotional abuse should be considered, given their relevance for well-being and mental health crises, as well as other forms of sexual violence. Additional open-ended questions may help capture subjective understandings of violence beyond predefined categories. Future studies may additionally examine weighted severity indices, cumulative exposure models, and repeated assessments to better capture the variability of interpersonal violence involvement over time. Moreover, future research should further explore adapted assessment approaches for individuals with acute psychosis or cognitive impairment. Multilingual versions may improve the inclusion of refugee and migrant populations at increased risk of violence exposure. Future studies may combine self-report and administrative data to compare prevalence estimates across data sources.

### Clinical implications

Standardized approaches for assessing interpersonal violence involvement are still limited in German psychiatric care and research settings. Various validated instruments are available, but they are typically limited to specific contexts, such as domestic and intimate partner violence risk assessment [59].

Routine assessment of interpersonal violence involvement in psychiatric settings may support comprehensive and trauma-informed psychiatric care. The IVIQ may support structured inquiry within regular clinical assessments of symptoms and stress factors, and may help to detect otherwise undisclosed victimization, given that psychiatric patients tend to under-report abuse in mental health care settings [60]. A standardized questionnaire may support patient autonomy in disclosing, thereby strengthening their agency, rather than leaving the decision whether to address these topics to clinicians. At the same time, professionals benefit from a clear and structured set of questions that can guide further therapeutic exploration and treatment planning.

Findings regarding social desirability underline that a trustworthy relationship between staff and patients remains crucial when assessing sensitive topics, including both violence victimization and perpetration. Repeated and routine assessment may further reduce bias and facilitate disclosure over time.

## Conclusion

The IVIQ is a structured self-report checklist for assessing patient-reported victimization and perpetration experiences in psychiatric populations. Findings provide preliminary support for the feasibility and acceptability of the IVIQ in psychiatric populations. Future development should consider broader conceptualizations of violence. Interpretation of self-reported violence experiences should consider potential influences of social desirability bias.

## Supplementary Information

Below is the link to the electronic supplementary material.

Supplementary Material 1

Supplementary Material 2

Supplementary Material 3

Supplementary Material 4

Supplementary Material 5

## Data Availability

The datasets generated and/or analysed during the current study are not publicly available due to privacy/ethical restrictions, but are available from the corresponding author on reasonable request.

## Abbreviations

IVIQ: Interpersonal Violence Questionnaire
SMI: Severe mental illness (SMI)
ICC: Intraclass Kappa Indices
KSE-G: Scale for assessing the social desirability trait (“Kurzskala Soziale Erwünschtheit-Gamma”)
