# Supplementary material for: Assessing interpersonal violence involvement in psychiatric patients: exploratory findings on the Interpersonal Violence Questionnaire (IVIQ)
Source: BMC Psychiatry. 2026 Sep 4;26:715. doi: 10.1186/s12888-026-08597-7 (PMC13563774; doi:10.1186/s12888-026-08597-7)
Supplement: Supplementary file 1 [file 12888_2026_8597_MOESM1_ESM.docx]

**Supplementary material 1. List of Clinics included in the recruitment process**

Leipzig University Hospital Clinic and Polyclinic for Psychiatry; Saxon Hospital Altscherbitz Clinic for Psychiatry, Psychotherapy, and Psychosomatics; Helios Park Clinic Leipzig; Bethanien Hochweitzschen Specialist Hospital, Center for Psychosocial Medicine; Psychiatry, Psychotherapy, and Psychosomatic Medicine, Helios Hanseklinikum Stralsund; The Clinic for Psychiatry and Psychotherapy Günzburg District Hospital, University Hospital Ulm; and The Psychosomatik Clinic University Hospital Ulm
