# Supplementary material for: Assessing interpersonal violence involvement in psychiatric patients: exploratory findings on the Interpersonal Violence Questionnaire (IVIQ)
Source: BMC Psychiatry. 2026 Sep 4;26:715. doi: 10.1186/s12888-026-08597-7 (PMC13563774; doi:10.1186/s12888-026-08597-7)
Supplement: Supplementary file 2 [file 12888_2026_8597_MOESM2_ESM.docx]

**Supplementary Table 2. List of Items, German and English**

This supplementary table presents the original German IVIQ items alongside their English translations for transparency and documentation of the translation/adaptation process.

|  |  | Item | | Type | | |
| --- | --- | --- | --- | --- | --- | --- |
| Past 12 months | (1) | vio_1 | Hat jemand einen Gegenstand nach Ihnen geworfen? (…has someone thrown something at you?) | vict | physical |  |
|  |  | vio_2 | Haben Sie selbst einen Gegenstand nach jemandem geworfen?  (…have you thrown something at someone?) | perp |  |  |
|  |  | vio_3 | Hat Sie jemand geschubst, gepackt oder gestoßen?  (…has anyone pushed, grabbed or shoved you?) | vict |  |  |
|  |  | vio_4 | Haben Sie selbst jemanden geschubst, gepackt oder gestoßen?  (…have you pushed, grabbed or shoved anyone?) | perp |  |  |
|  |  | vio_5 | Hat Sie jemand geohrfeigt?  (…has anyone slapped you?) | vict |  |  |
|  |  | vio_6 | Haben Sie selbst jemanden geohrfeigt?  (…have you slapped anyone?) | perp |  |  |
|  |  | vio_7 | Hat Sie jemand getreten, gebissen oder gewürgt?  (…has anyone kicked, bitten or choked you?) | vict |  |  |
|  |  | vio_8 | Haben Sie selbst jemanden getreten, gebissen oder gewürgt?  (…have you kicked, bitten or choked anyone?) | perp |  |  |
|  |  | vio_9 | Hat Sie jemand mit der Faust geschlagen oder verprügelt?  (…has anyone hit you with a fist or beaten you up?) | vict |  |  |
|  |  | vio_10 | Haben Sie selbst jemanden mit der Faust geschlagen oder verprügelt?  (…have you hit anyone with a fist or beaten up anyone?) | perp |  |  |
|  |  | vio_11 | Hat Ihnen jemand Gewalt angedroht?  (…has anyone threatened you with violence?) | vict | threat |  |
|  |  | vio_12 | Haben Sie selbst jemandem Gewalt angedroht?  (…have you threatened anyone with violence?) | perp |  |  |
|  | (2) higher severity | vio_13 | Hat jemand versucht, Sie gegen Ihren Willen zu sexuellen Handlungen zu zwingen?  (…has anyone tried to physically force you to have sex against your will?) | vict | sexual |  |
|  |  | vio_14 | Haben Sie versucht, jemanden gegen seinen/ihren Willen zu sexuellen Handlungen zu zwingen?  (…have you tried to physically force anyone to have sex against their will?) | perp |  |  |
|  |  | vio_15 | Hat Sie jemand mit einem Messer, einer Schusswaffe oder einer anderen Waffe bedroht?  (…has anyone threatened you with a knife, gun, or any other weapon?) | vict | threat |  |
|  |  | vio_16 | Haben Sie jemanden mit einem Messer, einer Schusswaffe oder einer anderen Waffe bedroht?  (…have you threatened anyone with a knife, gun, or any other weapon?) | perp |  |  |
|  |  | vio_17 | Hat jemand mit einer Waffe auf Sie geschossen oder ein Messer oder eine andere Waffe gegen Sie eingesetzt?  (…has anyone fired a gun at you or used a knife or any other weapon on you?) | vict | physical |  |
|  |  | vio_18 | Haben Sie mit einer Waffe auf jemanden geschossen oder ein Messer oder eine andere Waffe gegen ihn eingesetzt?  (…have you fired a gun at someone or used a knife or other weapon on them?) | perp |  |  |
|  |  | vio_19 | Haben Sie jemanden wirklich schwer verletzt? (nicht gemeint sind Verletzungen durch Unfälle)  (…have you hurt somebody really badly?) | perp |  |  |
| Lifetime | | vio_20 | Hat jemand versucht, Sie gegen Ihren Willen zu sexuellen Handlungen zu zwingen?  (…has anyone ever tried to physically force you to have sex against your will?) | vict | sexual |  |
|  |  | vio_21 | Haben Sie versucht, jemanden gegen seinen/ihren Willen zu sexuellen Handlungen zu zwingen?  (…have you ever tried to physically force anyone to have sex against their will?) | perp |  |  |
|  |  | vio_22 | Hat Sie jemand mit einem Messer, einer Schusswaffe oder einer anderen Waffe bedroht?  (…has anyone threatened you with a knife, gun, or any other weapon?) | vict | threat |  |
|  |  | vio_23 | Haben Sie jemanden mit einem Messer, einer Schusswaffe oder einer anderen Waffe bedroht?  (…have you threatened anyone with a knife, gun, or any other weapon?) | perp |  |  |
|  |  | vio_24 | Hat jemand mit einer Waffe auf Sie geschossen oder ein Messer oder eine andere Waffe gegen Sie eingesetzt?  (…has anyone fired a gun at you or used a knife or any other weapon on you?) | vict | physical |  |
|  |  | vio_25 | Haben Sie schon einmal mit einer Waffe auf jemanden geschossen oder ein Messer oder eine andere Waffe gegen ihn eingesetzt?  (…have you fired a gun at someone or used a knife or other weapon on them?) | perp |  |  |
|  |  | vio_26 | Haben Sie jemals jemanden wirklich schwer verletzt? (nicht gemeint sind Verletzungen durch Unfälle)  (…have you ever hurt somebody really badly?) | perp |  |  |
