# Supplementary material for: Assessing interpersonal violence involvement in psychiatric patients: exploratory findings on the Interpersonal Violence Questionnaire (IVIQ)
Source: BMC Psychiatry. 2026 Sep 4;26:715. doi: 10.1186/s12888-026-08597-7 (PMC13563774; doi:10.1186/s12888-026-08597-7)
Supplement: Supplementary file 3 [file 12888_2026_8597_MOESM3_ESM.docx]

**Supplementary Table 3. Test–retest agreement and ICC estimates for IVIQ items**

| **Item** | **n** | **Simple  agreement %** | **ICC (single)** | | **95%-CI** | | **F-Test** | | |
| --- | --- | --- | --- | --- | --- | --- | --- | --- | --- |
| *Past 12 months* |  |  |  | |  | |  | | |
| **vio_1**. Has someone thrown something at you? | 112 | 89.3 | .61 | | [.47, .71] | | 4.05*** | | |
| **vio_2.** Have you thrown something at someone? | 112 | 92.0 | .14 | | [-.05, .32] | | 1.33 | | |
| **vio_3.** Has anyone pushed, grabbed or shoved you? | 111 | 82.9 | .58 | | [.44, .69] | | 3.74*** | | |
| **vio_4.** Have you pushed, grabbed or shoved anyone? | 110 | 86.4 | .54 | | [.39, .66] | | 3.45*** | | |
| **vio_5.** Has anyone slapped you? | 112 | 88.4 | .37 | | [.20, .52] | | 2.18*** | | |
| **vio_6**. Have you slapped anyone? | 111 | 92.8 | .39 | | [.22, .54] | | 2.29*** | | |
| **vio_7.** Has anyone kicked, bitten or choked you? | 109 | 89.9 | .47 | | [.31, .60] | | 2.74*** | | |
| **vio_8.** Have you kicked, bitten or choked anyone? | 111 | 93.7 | -.03 | | [-.22, .16] | | 0.94 | | |
| **vio_9.** Has anyone hit you with a fist or beaten you up? | 111 | 87.4 | .35 | | [.18, .50] | | 2.08*** | | |
| **vio_10.** Have you hit anyone with a fist or beaten up anyone? | 110 | 91.8 | .14 | | [-.05, .32] | | 1.32 | | |
| **vio_11.** Has anyone threatened you with violence? | 101 | 75.2 | .43 | | [.25, .57] | | 2.47*** | | |
| **vio_12.** Have you threatened anyone with violence? | 111 | 90.1 | .54 | | [.39, .66] | | 3.31*** | | |
| **vio_13.** Has anyone tried to physically force you to have sex against your will? | 105 | 93.3 | .71 | | [.59, .79] | | 5.79*** | | |
| **vio_14.** Have you tried to physically force anyone to have sex against their will? | 113 | 100.0 | - | | - | | - | | |
| **vio_15.** Has anyone threatened you with a knife, gun, or any other weapon? | 112 | 89.3 | .44 | | [.28, .58] | | 2.58*** | | |
| **vio_16.** Have you threatened anyone with a knife, gun, or any other weapon? | 113 | 95.6 | -.02 | | [-.21, .16] | | 0.96 | | |
| **vio_17.** Has anyone fired a gun at you or used a knife or any other weapon on you? | 113 | 96.5 | -.02 | | [-.20, .17] | | 0.96 | | |
| **vio_18.** Have you fired a gun at someone or used a knife or other weapon on them? | 112 | 98.2 | -.01 | | [-.20, .18] | | 0.98 | | |
| **vio_19**. Have you hurt somebody really badly? | 107 | 95.3 | -.02 | | [-.21, .17] | | 0.96 | | |
| *Lifetime* |  |  |  | |  | |  | | |
| **vio_20**. Has anyone ever tried to physically force you to have sex against your will? | 109 | 83.5 | .67 | | [.55, .76] | | 5.11*** | | |
| **vio_21.** Have you ever tried to physically force anyone to have sex against their will? | 111 | 98.2 | .74 | | [.65, .82] | | 6.83*** | | |
| **vio_22.** Has anyone threatened you with a knife, gun, or any other weapon? | 109 | 80.7 | .49 | | [.33, .62] | | 3.05*** | | |
| **vio_23**. Have you threatened anyone with a knife, gun, or any other weapon? | 107 | 96.3 | .65 | | [.53, .75] | | 4.71*** | | |
| **vio_24.** Has anyone fired a gun at you or used a knife or any other weapon on you? | 108 | 90.7 | | .32 | | [.15, .49] | | 1.96*** |  |
| **vio_25.** Have you fired a gun at someone or used a knife or other weapon on them? | 109 | 97.2 | .00 | | [-.18, .19] | | 1.00 | | |
| **vio_26.** Have you ever hurt somebody really badly? | 109 | 94.5 | .67 | | [.55, .76] | | 5.06*** | | |

**Note.** Simple agreement indices based on n = number of complete T1–T2 pairs. ICC = intraclass correlation coefficient (two-way mixed-effects, absolute agreement, single measures). Item 14 showed no variance; ICC could not be estimated
