# Supplementary material for: Assessing interpersonal violence involvement in psychiatric patients: exploratory findings on the Interpersonal Violence Questionnaire (IVIQ)
Source: BMC Psychiatry. 2026 Sep 4;26:715. doi: 10.1186/s12888-026-08597-7 (PMC13563774; doi:10.1186/s12888-026-08597-7)
Supplement: Supplementary file 4 [file 12888_2026_8597_MOESM4_ESM.docx]

**Supplementary material 4. Original German free-text comments provided by participants**

**Note.** Free-text responses are presented in their original German wording to preserve meaning and contextual nuances. The survey was conducted exclusively in German; therefore, no official English translations of participant quotations are provided.

Free-text responses were analyzed descriptively and grouped into thematic categories relevant to the interpretation and future development of the IVIQ.

Participant free-text comments were grouped thematically into the following categories:

A. Comments related to questionnaire acceptability, timeframe coverage, and memory-related difficulties

B. Comments related to psychological, emotional, and verbal violence

C. Comments highlighting the perceived relevance of the research topic and disclosure in psychiatric care

A. Comments related to questionnaire acceptability, timeframe coverage, and memory-related difficulties

“Do you have any comments or thoughts about violence you have experienced or enacted?”
(org.: “Haben Sie noch Anmerkungen oder Gedanken zur Gewalt, die Sie erlebt/ausgeübt haben?“)

Violence Victimization

„als Kind Gegenstände nach mir geworfen bekommen“
„häusliche Gewalt durch Mutter und Geschwister sowie in der Schule. Als Kind und als Jugendlicher.“
„Waffenfreie Gewalt in der Kindheit“
„es waren nicht nur Vergewaltigungen, sondern auch Prügel durch die Mutter“
„Habe in meiner Kindheit ca. bis zum 16. L.J. den Po versohlt bekommen .“
„Ich wurde geschlagen in der Kindheit von meinem Stiefvater, Straße + damals zu Hause“
„Habe es im vorherigen Fragebogen vielleicht übersehen, aber ich wurde als Kind manchmal von meinen Eltern geschlagen (Ohrfeigen)“
„Ich habe teilweise Lücken in der Erinnerung zu sexueller Gewalt aus meiner Kindheit und kann die genauere Anzahl nicht nennen.“
„Wurde als Kind geschlagen.“
„Sehr viel verbale Gewalt, gezieltes Training auf Verhaltensweisen, körperliche Gewalt, Zwang, Bestrafungen, Drohungen, absichtlich etwas unbesiegbar machen damit eine Bestrafung folgen kann, Schlafentzug, Entzug aus sozialem Umfeld“
„Ein großes Thema betrifft die häusliche Gewalt innerhalb der Familie. Meistens sind es Männer / Väter, allem voran, wenn verstärkter Alkoholkonsum im Spiel besteht…“

Violence Perpetration

-

“Do you have any comments on the questionnaire or on these topics in general?”
(org.: “Haben Sie Anmerkungen zum Fragebogen oder generell zu diesen Themen?“)

“Wieso so viele Fragen doppelt?”
„Ich hätte es wichtig gefunden, wenn alle Fragen von den letzten 12 Monaten auch bei den Erfahrungen des gesamten Lebens verwendet worden wären.“
„Bei 12M eingetragen, bezieht sich aber auf ganzes Leben:….“
„Ja, das Problem ist bei mir, dass ich so viel verdrängt habe, dass manche Fragen im Nachhinein gewirkt haben und mir im Nachhinein erst Dinge eingefallen sind. Man müsste solche Fragebögen regelmäßig durchführen.“
„Gar nichts zu häuslicher Gewalt, etwas überraschend“
„Fragen teilweise zu verstrickt gestellt“
„Manche Fragestellungen sind etwas komisch formuliert, da manche Antworten einfach wiederholt werden.“
„So schwere Gewalt habe ich nicht erfahren, aber leichtere“
„Ich habe in meiner Kindheit oft häusliche Gewalt in Form von Schlagen (mit und ohne Gegenstände/ gegen mich und andere Familienmitglieder erlebt)“
„Ich finde, es liegt im Ermessen von Gewalt betroffenen Personen, diese zu benennen. Inwiefern andere Menschen Verhaltensweisen oder Aussagen von mir als gewaltvoll wahrgenommen haben, die mir aber nicht mitgeteilt haben, macht die Einschätzung schwierig. Ich glaube nicht, dass es Menschen gibt, die komplette Gewaltfreiheit erleben oder ausführen.“
„Es fehlen Fragen zur psychischen Gewalt“
„Psychische Gewalt ist viel schlimmer für mich als körperliche“
„Psychische Gewalt hab ich definitiv viel mehr erfahren müssen als körperliche“
„Uneindeutige Androhungen“
„Die schlimmsten Erfahrungen hatte ich mit meinem ehemaligen Stiefvater; er hat mich oft angeschrien, Sachen nach mir geworfen und mir Gewalt angedroht.“
„Psychische Gewalt wird nicht geahndet - keine Zeugen = alles gelogen - Menschen, die psychische Gewalt erlebt haben, erleben auch später diese - Beziehung als Trauma --> sind ihr Leben lang gezeichnet, müssen eine Therapie machen, während die Tä“
„Schläge und emotionale Erpressung durch die Eltern in meiner Kindheit“
„ausgeübte Gewalt: seelische Verletzungen“
„Es gibt psychische und körperliche Gewalt“
„Was ist mit emotionaler Gewalt“
„verbale Androhungen von Gewalt vom Vater - verbale sexuelle Belästigung auf der Arbeit“
„verbale Gewalt durch Vater“
„Psychische Gewalt war auch dabei“
„Indirekte Gewalt, Naivität“
„berufliche Gewalt am Arbeitsplatz, von Chef überwiegend ignoriert (2022 in Berufg von einem Kollegen verbale und körperliche Gewalt)“
„Psychische Gewalt könnte noch aufgenommen werden“
„Ich habe psychische Gewalt erlebt“
„Gewalt muss nicht zwangsläufig körperlich sein. Worte und indirekte Drohungen oder Anspielungen können ebenso schmerzhaft sein.
„seelische Gewalt tut weh“
„Psychische Gewalt kommt zu kurz.“
„Habe keine körperliche Gewalt erlebt sondern seelische Gewalt“
„Was ist mit Gewalterfahrungen durch Unterlassung? Z. B. von Erziehenden Kindern gegenüber? Was ist mit Mobbing? Was ist mit sexualisierter Gewalt?“
„verbale Beschimpfungen, Manipulationen, narzistischer Missbrauch“
„Verbale Gewalt durch meinen Vater und Tritte von ihm“
„Als Kind keine direkte Gewalt sonder psychische Gewalt erfahren“
„Es gibt nicht nur köperliche Gewalt“
„Ich bin etwas irritiert von der Einteilung des Fragebogens in Gewalterfahrungen, die scheinbar nur körperliche
Gewalt bedeuten, und negative Erfahrungen, die damit scheinbar nicht als Gewalt bezeichnet werden. Das ist meines Erachtens nicht nur falsch, sondern reproduziert auch Formen von Gewalt, indem es sie unsichtbar macht. Ist es Interesse der Studie, nur Erfahrungen zu körperlicher Gewalt zu sammeln, so sollte das benannt werden. So habe ich das Gefühl, meine Gewalterfahrungen finden in dieser Studie nur Raum in den Anmerkungen und das fühlt sich falsch an“
„Negative Erfahrungen gehen über direkte Angriffe und böse Absichten hinaus. Falsch gegendert zu werden z.B. ist selten böse gemeint und eher das Versäumnis einer binären Sprache und binären Kategorien, mit denen wir alle wachsen. Das Privileg, sich damit nicht auseinandersetzen zu müssen, führt bisher oft zu Ignoranz und fehlender Empathie. Trotzdem ist es für mich eine gewaltvolle Erfahrung, in einer binären Gesellschaft zu leben, ständig falsch angesprochen, und damit eingeordnet, zu werden und allzu oft verantwortlich dafür gemacht zu werden, Bildungsarbeit zu leisten und meinen Mitmenschen fehlerfreundlich zu begegnen und ihnen in ihrer Ignoranz oder Überforderung ein gutes Gefühl zu geben. Dazu kommt die ständige Angst, Gewalt zu erfahren.“
„psychische Gewalt“
„Viele erleben neben physischer Gewalt vor allem psychische Gewalt. Es ist Schade, dass darauf keinen Bezug genommen wurde“
„Die meiste Gewalt die ich erlebt habe war psychischer Natur oder physisch zu Schulzeiten“
„Es sollte auch mehr über psychische Gewalt gesprochen werden. Die ist leider nie sichtbar und passiert noch häufiger.“
„Ich habe viel Psychische Gewalt erlebt, fühle mich als narzistisches Missbrauchsopfer“
„Ich bin der Meinung, dass psychischer Terror und Grausamkeit auch härter geahndet werden müssten.“
„Zur Physischen kam psychische Gewalt, die einem alles nehmen kann (misstraut eigenen Gedanken, Gefühlt Abhängigkeit steigt, Selbstwert sinkt, Machtverhältnis durch Peiniger ausgebaut“
„Sehr viel verbale Gewalt, gezieltes Training auf Verhaltensweisen, körperliche Gewalt, Zwang, Bestrafungen, Drohungen, absichtlich etwas unbesiegbar machen damit eine Bestrafung folgen kann, schlafentzug, Entzug aus sozialem umfeld“
„Traumata,angst erstickt,geschlagen oder gepackt zu werden,Selbsthass,Angst vor meinem leiblichen Vater“
„Primär nutze ich verbale Gewalt, da ich mit Worten besser verletzen kann als mit meiner Faust.“
„In meiner Kindheit habe ich oft psychische Gewalt erlebt.“
„Ich denke, dass die ehemalige Freundin meine schlechte psychische Verfassung ausnutze, um sexuelle Handlungen zu betreiben. Ich wollte das nicht, konnte es aber nicht äußern.“
„psychische Gewalt durch Stiefvater“
„Ich finde es gut, wenn die Ärzte den Fragebogen auch lesen dürfen.“
„Nein, alles in Ordnung. Finde ich gut, dass solche Studien durchgeführt werden.“
„gut, dass es FB gibt“
„Ich wünsche mir, dass über das Thema Gewalt viel mehr gesprochen werden muss! Und es dafür Anlaufstellen geben sollte. Viel bleibt im Verborgenen. Dankeschön!“
„Ich finde es gut, dass eine Studie zu diesem Thema in die Wege geleitet wird und in diesem Sinne mit dem Fragebogen wichtige Informationen gesammelt werden. Sehr freundlich, wie die Mitarbeiterinnen im Rahmen des Fragebogens auf die Teilnehmer zugehen und erklären. Ich hoffe, es trägt dazu bei, die Stigmatisierung von Menschen mit psychischen Erkrankungen abzubauen.“

B. Comments related to psychological, emotional, and verbal violence

C. Comments highlighting the perceived relevance of the research topic and disclosure in psychiatric care
