# Supplementary material for: Assessing interpersonal violence involvement in psychiatric patients: exploratory findings on the Interpersonal Violence Questionnaire (IVIQ)
Source: BMC Psychiatry. 2026 Sep 4;26:715. doi: 10.1186/s12888-026-08597-7 (PMC13563774; doi:10.1186/s12888-026-08597-7)
Supplement: Supplementary file 5 [file 12888_2026_8597_MOESM5_ESM.docx]

**Supplementary Table 5. Inter-Item Phi-coeff correlation matrix**

|  | vio_3 | vio_4 | vio_1 | vio_2 | vio_5 | vio_6 | vio_7 | vio_8 | vio_9 | vio_10 | vio_11 | vio_12 | vio_13 | vio_14 | vio_15 | vio_16 | vio_17 | vio_18 | vio_19 | vio_20 | vio_21 | vio_22 | vio_23 | vio_24 | vio_25 | vio_26 |
| --- | --- | --- | --- | --- | --- | --- | --- | --- | --- | --- | --- | --- | --- | --- | --- | --- | --- | --- | --- | --- | --- | --- | --- | --- | --- | --- |
| vio_3 | 1 | *** | *** | *** | *** | *** | *** | *** | *** | *** | *** | *** | *** |  | *** | ** | ** |  | *** | * |  | *** |  | ** |  |  |
| vio_4 | 0,47 | 1 | *** | *** | *** | *** | *** | *** | *** | *** | *** | *** | * |  | *** | *** |  |  | *** |  |  | *** |  |  |  | *** |
| vio_1 | 0,58 | 0,34 | 1 | *** | *** | *** | *** | *** | *** | *** | *** | ** | *** |  | *** | * | *** |  | *** | *** |  | *** |  | *** |  | ** |
| vio_2 | 0,24 | 0,35 | 0,33 | 1 | *** | * | *** | *** | ** | *** | *** | *** |  | * | *** | *** |  |  | *** |  |  | ** | * |  |  | *** |
| vio_5 | 0,54 | 0,41 | 0,53 | 0,21 | 1 | *** | *** | *** | *** | *** | *** | *** | *** |  | *** | ** | *** |  | *** |  |  |  |  | *** |  | *** |
| vio_6 | 0,32 | 0,45 | 0,26 | 0,18 | 0,39 | 1 | *** | *** | *** | *** | *** | *** | ** | * | *** | *** | *** |  | *** |  |  |  |  |  |  | *** |
| vio_7 | 0,56 | 0,39 | 0,56 | 0,26 | 0,55 | 0,3 | 1 | *** | *** | *** | *** | *** | *** | *** | *** | *** |  |  | *** |  |  | *** | ** |  |  | *** |
| vio_8 | 0,3 | 0,39 | 0,26 | 0,33 | 0,33 | 0,37 | 0,37 | 1 | *** | *** | *** | *** | *** | *** | *** | *** |  |  | *** |  |  | *** | ** |  |  | *** |
| vio_9 | 0,44 | 0,3 | 0,52 | 0,18 | 0,52 | 0,25 | 0,56 | 0,22 | 1 | *** | *** | *** | *** |  | *** | *** | *** |  | *** |  |  | *** | ** | *** |  | ** |
| vio_10 | 0,3 | 0,43 | 0,31 | 0,24 | 0,36 | 0,48 | 0,27 | 0,43 | 0,42 | 1 | *** | *** |  | ** | *** | *** | *** |  | *** |  |  | *** | *** | *** | ** | *** |
| vio_11 | 0,55 | 0,3 | 0,45 | 0,28 | 0,41 | 0,29 | 0,41 | 0,24 | 0,41 | 0,34 | 1 | *** | *** |  | *** | *** | *** |  | *** | * |  | ** |  |  |  | *** |
| vio_12 | 0,27 | 0,46 | 0,2 | 0,29 | 0,28 | 0,32 | 0,26 | 0,32 | 0,32 | 0,53 | 0,37 | 1 |  |  | *** | *** | *** |  | *** |  |  | *** | *** | *** | *** | *** |
| vio_13 | 0,38 | 0,17 | 0,41 | 0,1 | 0,35 | 0,19 | 0,45 | 0,22 | 0,34 | 0,07 | 0,37 | 0,09 | 1 |  | *** |  | *** |  |  | *** |  | ** |  | * |  |  |
| vio_14 | 0,08 | 0,1 | 0,1 | 0,17 | 0,11 | 0,17 | -0,02 | 0,21 | 0,14 | 0,19 | 0,07 | 0,13 | 0,12 | 1 |  | *** | *** | *** | ** |  | *** |  |  |  | *** |  |
| vio_15 | 0,42 | 0,33 | 0,49 | 0,27 | 0,46 | 0,33 | 0,51 | 0,33 | 0,51 | 0,43 | 0,38 | 0,33 | 0,36 | 0,14 | 1 | *** | *** |  | *** |  |  | *** | *** | *** | *** | *** |
| vio_16 | 0,19 | 0,27 | 0,18 | 0,29 | 0,2 | 0,38 | 0,2 | 0,36 | 0,23 | 0,4 | 0,25 | 0,39 | 0,07 | 0,25 | 0,38 | 1 |  | *** | *** |  |  | *** | *** |  | *** | *** |
| vio_17 | 0,21 | 0,13 | 0,34 | 0,07 | 0,36 | 0,25 | 0,23 | 0,11 | 0,4 | 0,35 | 0,22 | 0,23 | 0,22 | 0,23 | 0,47 | 0,15 | 1 | *** | *** |  |  | *** |  | *** | *** |  |
| vio_18 | 0,1 | 0,05 | 0,1 | 0,04 | 0,12 | 0,12 | 0,06 | 0,15 | 0,15 | 0,13 | 0,14 | 0,14 | 0,13 | 0,41 | 0,15 | 0,21 | 0,29 | 1 |  |  |  | * | *** | *** | *** | * |
| vio_19 | 0,3 | 0,34 | 0,26 | 0,26 | 0,39 | 0,35 | 0,34 | 0,43 | 0,28 | 0,45 | 0,26 | 0,39 | 0,1 | 0,19 | 0,33 | 0,36 | 0,25 | 0,15 | 1 |  |  | ** | *** | *** | *** | *** |
| vio_20 | 0,18 | 0,03 | 0,21 | 0,04 | 0,09 | 0,02 | 0,14 | 0,05 | 0,09 | -0,03 | 0,18 | 0,02 | 0,41 | 0,05 | 0,13 | -0,04 | 0,1 | 0,13 | -0,05 | 1 |  | *** |  |  |  |  |
| vio_21 | -0,02 | -0,03 | 0,01 | 0,02 | 0,02 | 0,02 | -0,02 | 0,04 | 0,05 | 0,03 | 0 | 0,04 | 0,04 | 0,31 | 0,05 | 0,06 | 0,12 | 0,11 | 0,09 | 0,07 | 1 | *** | * |  | * |  |
| vio_22 | 0,24 | 0,24 | 0,23 | 0,19 | 0,15 | 0,16 | 0,24 | 0,26 | 0,29 | 0,25 | 0,21 | 0,23 | 0,2 | 0,09 | 0,42 | 0,23 | 0,27 | 0,17 | 0,19 | 0,21 | 0,21 | 1 | *** | *** | *** | *** |
| vio_23 | 0,12 | 0,13 | 0,13 | 0,17 | 0,1 | 0,11 | 0,12 | 0,21 | 0,21 | 0,24 | 0,09 | 0,31 | 0,07 | 0,16 | 0,27 | 0,4 | 0,15 | 0,29 | 0,26 | 0,01 | 0,18 | 0,38 | 1 | *** | *** | *** |
| vio_24 | 0,18 | 0,12 | 0,21 | 0,02 | 0,22 | 0,13 | 0,2 | 0,13 | 0,24 | 0,21 | 0,16 | 0,24 | 0,18 | 0,14 | 0,32 | 0,1 | 0,42 | 0,27 | 0,23 | 0,15 | 0,14 | 0,54 | 0,41 | 1 | *** | *** |
| vio_25 | 0,08 | 0,1 | 0,1 | 0,06 | 0,12 | 0,14 | 0,06 | 0,15 | 0,14 | 0,2 | 0,07 | 0,25 | 0,1 | 0,21 | 0,24 | 0,25 | 0,22 | 0,43 | 0,24 | 0,07 | 0,18 | 0,36 | 0,52 | 0,5 | 1 | *** |
| vio_26 | 0,16 | 0,24 | 0,19 | 0,21 | 0,22 | 0,23 | 0,2 | 0,32 | 0,21 | 0,36 | 0,22 | 0,34 | 0,03 | 0,13 | 0,27 | 0,36 | 0,16 | 0,16 | 0,51 | 0,01 | 0,09 | 0,3 | 0,36 | 0,31 | 0,36 | 1 |

**Items**

*Past 12 Months*

**vio_1**. Has someone thrown something at you?

**vio_2.** Have you thrown something at someone?

**vio_3.** Has anyone pushed, grabbed or shoved you?

**vio_4.** Have you pushed, grabbed or shoved anyone?

**vio_5.** Has anyone slapped you?

**vio_6**. Have you slapped anyone?

**vio_7.** Has anyone kicked, bitten or choked you?

**vio_8.** Have you kicked, bitten or choked anyone?

**vio_9.** Has anyone hit you with a fist or beaten you up?

**vio_10.** Have you hit anyone with a fist or beaten up anyone?

**vio_11.** Has anyone threatened you with violence?

**vio_12.** Have you threatened anyone with violence?

**vio_13.** Has anyone tried to physically force you to have sex against your will?

**vio_14.** Have you tried to physically force anyone to have sex against their will?

**vio_15.** Has anyone threatened you with a knife, gun, or any other weapon?

**vio_16.** Have you threatened anyone with a knife, gun, or any other weapon?

**vio_17.** Has anyone fired a gun at you or used a knife or any other weapon on you?

**vio_18.** Have you fired a gun at someone or used a knife or other weapon on them?

**vio_19**. Have you hurt somebody really badly?

*Lifetime*

**vio_20**. Has anyone ever tried to physically force you to have sex against your will?

**vio_21.** Have you ever tried to physically force anyone to have sex against their will?

**vio_22.** Has anyone threatened you with a knife, gun, or any other weapon?

**vio_23**. Have you threatened anyone with a knife, gun, or any other weapon?

**vio_24.** Has anyone fired a gun at you or used a knife or any other weapon on you?

**vio_25.** Have you fired a gun at someone or used a knife or other weapon on them?

**vio_26.** Have you ever hurt somebody really badly?
